# Quality of life on hemodialysis and inflammation: a descriptive analysis

**DOI:** 10.1590/1414-431X20187355

**Published:** 2018-04-19

**Authors:** M.R. Bacci, F. Adami, F.W.S. Figueiredo, B.C.A. Alves, G.L. da Veiga, F.L.A. Fonseca

**Affiliations:** 1 Departamento de Clínica Médica, Faculdade de Medicina ABC, Santo André, SP, Brasil; 2 Laboratório de Análises Clínicas, Faculdade de Medicina ABC, Santo André, SP, Brasil; 3 Laboratório de Epidemiologia e Análises de Dados, Faculdade de Medicina ABC, Santo André, SP, Brasil; 4 Departamento de Ciências Farmacêuticas, Universidade Federal de São Paulo, Diadema, SP, Brasil

**Keywords:** Quality of life, Hemodialysis, Inflammation, Homocysteine

## Abstract

Chronic kidney disease (CKD) is highly prevalent worldwide. Patients with CKD on hemodialysis are more likely to present behavioral changes and worse quality of life as a result of their routine and complications. They also have higher levels of cytokines. The aim of this study is to assess the relationship between the inflammatory profile and quality of life measured by KDOQL-SF36 in hemodialysis outpatients. Patients older than 21 years of age and on routine hemodialysis for at least 6 months with treatment on a regular weekly basis were included and their anthropometric parameters and serum inflammatory markers were evaluated. Thirty patients consented to participate. Homocysteine (Hcy) levels were correlated with worse glomerular filtration rate (GFR; P=0.003) and creatinine (P=0.002). IL-6 was not correlated with worse nutritional status taking into account body mass index (BMI; kg/m^2^; P=0.83). On the other hand, TNF-alpha was positively correlated with albumin (P=0.008), nutritional status by BMI (P=0.04), and nutritional status by arm circumference area (P=0.04). IL-6 was correlated with activity limitation (P=0.02) and Hcy with work status (P=0.04). Hcy was correlated with nutritional status and inflammatory markers. In this population, the majority of the sections in KDOQL-SF36 were not correlated with cytokines levels.

## Introduction

The terminal stage of chronic kidney disease (CKD) is characterized by a change in the routine of the individual when dialysis treatment begins. Quality of life assessments through the application of validated questionnaires is a simple and important tool to measure the quality of treatment and the individual's acceptance of the disease (1,2).

Kidney Disease and Quality-of-Life Short-Form 36 (KDQOL-SF36) is a quality-of-life assessment tool for patients with CKD undergoing hemodialysis and peritoneal dialysis, able to subjectively assess some parameters of the patient's daily life that can reflect on their well-being and quality of life. The questionnaire is self-applicable and has a multi-item scale that evaluates health concepts, ranging from limitations on physical activities and health problems, social limitations due to physical and emotional problems, limitations in day-to-day activities due to health problems, pain, and mental health among others (3).

Dialysis patients may present a progressive reduction of their cognitive and intellectual level, nutritional alterations, a higher propensity to infections, and worsening of quality of life, although an improvement of clinical and laboratory conditions has been described (4). One of the hypotheses for changes in cognitive function in CKD patients is the increase in the synthesis of pro-inflammatory mediators (1,4,5). Higher levels of proinflammatory cytokines and other mediators of inflammation have been considered potential agents that favor the reduction of quality of life in patients with CKD on dialysis (5). It is common for patients in this clinical condition to present endotoxemia and this, in parallel, further exacerbates renal dysfunction (6).

In addition, the release of inflammatory mediators during hemodialysis treatment is indicated as an aggravating factor for the increase in morbidity, mortality, and decline in the subjective perception of overall quality of life (7). In our study, we chose inflammatory markers that had already been described in other studies. Saraheimo et al. (8) demonstrated a close relationship between increased IL-6 levels and C-reactive protein parallel to the severity of renal disease. The augment of this cytokine is gradual and according to the progress of CKD (9). Homocysteine (Hcy) is a thrombogenic factor that antagonizes nitric oxide. Besides, hyperhomocysteinemia represents a risk factor for cardiovascular diseases and mortality in patients with CKD, especially when associated with high levels of CRP (10).

This information raised the need to search for possible correlations between the biochemical and inflammatory changes present in terminal CKD and some parameters of the KDQOL-SF36 questionnaire. The aim of the present study was to apply the KDQOL-SF36 adapted to the Portuguese language and to observe its association with inflammatory and biochemical markers.

## Material and Methods

### Patients

This was an analytical and descriptive observational study. All patients on hemodialysis treatment in Hospital Mário Covas and Centro de Hemodiálise de Mauá (SP, Brazil) were invited to take part in this study.

Patient inclusion criteria were: a) age greater than 21 years; b) confirmed diagnosis of end-stage CKD; and c) having had 3 hemodialysis sessions per week for at least 6 months.

The exclusion criteria were: a) systemic infection or hospitalization at the time of the interview; b) recent diagnosis or undergoing treatment for neoplasia; and c) psychiatric illness under treatment at the time of the interview. No patients were excluded from the study; all the interviewees met the inclusion criteria and thirty patients, after giving their written informed consent, answered the KDQOL-SF36 validated for the Portuguese language during the dialysis sessions. The survey was registered with the local Ethics Committee (No. 19795613.6.0000.0082/2013).

### Variables

Blood samples were collected only once at the start of the hemodialysis session on the day of the interview. The inflammatory markers used were Hcy, ultra-sensitive C-reactive protein (CRP), interleukin 6 (IL-6), TNF-α, and serum ferritin. The adequacy assessment of the hemodialysis was performed through the single pool kt/v value of each session on the day of the interview. A value above 1.2 was considered a reference for adequacy.

For the estimation of glomerular filtration rate (GFR), the simplified Modification of Diet in Renal Disease formula was used and the result reported in mL·min^−1^·(1.73 m^2^)^−1^.

The anthropometric measurements of each patient were performed by a single nutritionist using triceps skinfold, abdominal circumference, arm muscle circumference, and body mass index (BMI) (kg/m^2^). The weight used was the ideal dry weight in kilograms. The nutritional status of each patient was also assessed by the serum albumin value.

### Statistical analysis

All variables were analyzed descriptively. For quantitative variables, analysis was performed by observing the minimum and maximum values, and the calculation of means, SD, and medians. For qualitative variables, absolute and relative frequencies were calculated.

Using the Shapiro-Wilk test, it was verified that the majority of the data did not present a normal distribution (P<0.05) and were therefore reported as medians and interquartile ranges. To analyze the association between the variables, the Spearman correlation test was used. Statistical significance was set at P≤0.05. The statistical package used was Stata version 12.0 (StataCorp LP, USA).

## Results

The percentage and average values of the demographic, anthropometric, and nutritional characteristics of the patients are reported in Table 1.

**Table 1.** Demographic, anthropometric, and nutritional characteristics of the study population.

Variable	Value
Gender (M/F; %)	45/55
Age (years; average)	41.03
Ethnic group (Blacks/Caucasians; %)	43.3/56.4
Time in hemodialysis (months; average)	52.5
Kt/V (average)	1.34
Arterial hypertension (%)	93.3
Diabetes (%)	23.3
Etiology of chronic kidney disease (%)	
Arterial hypertension	43.5
Diabetes	13.3
Glomerulonephritis	10.0
Autoimmune	3.3
Undefined	26.6
Chronic pyelonephritis	3.3
Interdialytic gain (%)	
Up to 3 kg	53.4
From 3 to 5 kg	36.6
Above 5 kg	10.0
Nutritional status (%)	
Malnourished	3.4
Eutrophic	46.6
Overweight	40.0
Obese	10.0

Data are reported as percentages or averages.

The KDQOL-SF36 data of the items of interest are reported in Table 2 as medians (25–75th percentiles).

**Table 2.** Medians for items of the kidney disease quality of life short form 36 (KDQOL-SF 36) questionnaire.

Quality of life	Median (25–75%)
Chronic renal failure – specific domains	
Patient satisfaction	5.5 (5–6)
Dialysis staff encouragement	4 (2–6)
Social support	7 (6–8)
Sleep	16 (13–18)
Sexual function	2 (2–3)
Quality of social interactions	9 (7–10)
Cognitive function	5 (3–7)
Work status	3 (3–3)
Burden of kidney disease	14 (11–16)
Effects of kidney disease	18 (13–20)
List of symptoms/problems	16.5 (14–25)
Total	100
KDQOL-SF-36	
Mental health	19 (17–21)
Limitations due to emotional health problems	5.5 (4–6)
Social functioning	6 (6–6)
Energy/Fatigue	15 (17–13)
Pain	3 (2–4)
Perception of general health	17 (19–13)
Physical functioning	27 (24–28)
Limitations due to physical health problems	7 (6–8)
Total	99.5

Among the inflammatory markers studied, the highest value was for ferritin, with a median of 305.5 ng/mL (224–380). The medians for the other markers were TNF-α = 16.7 pg/mL (12.9–28.30), Hcy = 13.5 μmol/L (10.7–18.1), IL-6 = 12.5 pg/mL (5–18), and CRP = 12.5 mg/L (6–20).

Evaluation of anthropometric parameters was made through the measurement of abdominal circumference, body mass index (BMI), triceps skinfold, and arm circumference. For these parameters, the following medians were obtained: abdominal circumference of 90.5 cm (75–96); BMI of 25.2 kg/height (m)^2^ (21.5–27.3); triceps skinfold thickness of 20 cm (15–25); and arm circumference of 22.3 cm (21.2–24.4).

Statistical correlations between Hcy levels and work status and between IL-6 levels and limitations due to health problems were found. There was no relationship between the other indices evaluated (Table 3).

**Table 3.** Spearman’s correlation coefficients between quality of life and inflammatory markers.

Quality of life	Inflammatory markers				
	TNF-α	IL-6	CRP	Hcy	Ferritin
Chronic renal failure – specific domains					
Patient satisfaction	0.165	0.083	−0.156	−0.052	0.026
Dialysis staff encouragement	−0.146	−0.034	0.358	0.053	0.214
Social support	0.014	0.108	0.092	−0.016	−0.105
Sleep	0.234	−0.132	−0.116	−0.315	0.190
Sexual function	−0.182	0.038	−0.132	0.031	−0.031
Quality of social interactions	−0.036	−0.110	−0.038	0.053	0.100
Cognitive function	0.075	−0.004	0.084	−0.279	0.033
Work status	0.114	0.309	0.259	−0.374*	−0.106
Burden of kidney disease	0.171	0.075	−0.019	−0.346	0.055
Effects of kidney disease	0.123	0.161	0.300	0.061	0.328
List of symptoms/problems	0.032	0.064	0.202	−0.026	0.144
KDQOL-SF 36					
Mental health	−0.130	−0.319	0.008	0.264	0.110
Limitations due to emotional health problems	−0.026	−0.180	−0.172	−0.277	−0.001
Social functioning	0.023	0.268	0.313	−0.136	−0.007
Energy/Fatigue	0.210	−0.002	0.214	0.228	−0.007
Pain	−0.035	0.084	0.184	0.042	0.061
Perception of general health	−0.161	0.108	0.205	0.021	0.022
Physical Functioning	−0.174	−0.216	−0.058	0.018	−0.005
Limitations due to physical health problems	0.358	0.404**	−0.147	−0.266	0.023

Data are reported as Spearman’s correlation coefficient (rho). TNF-α: tumor necrosis factor alpha; IL-6: interleukin 6; CRP: ultra-sensitive C-reactive protein; Hcy: homocysteine; KDQOL-SF 36: kidney disease quality of life short form 36 questionnaire. *P<0.05; **P<0.01.

Associations were found between triceps skinfold thickness and work status (P=0.01), pain and triceps skinfold (P=0.05), and between arm circumference and patient satisfaction (P=0.03) and physical functioning (P=0.04) (Table 4).

**Table 4.** Spearman’s correlation coefficients between quality of life and anthropometric parameters.

Quality of life	Anthropometric parameters			
	Abdominal circumference	BMI	Triceps skinfold	Arm circumference
Chronic renal failure – specific domains				
Patient satisfaction	−0.254	−0.136	0.124	−0.378*
Dialysis staff encouragement	0.302	0.114	−0.311	0.318
Social support	0.033	−0.161	0.253	−0.322
Sleep	−0.231	−0.150	−0.204	−0.053
Sexual function	−0.193	−0.298	−0.325	−0.085
Quality of social interactions	−0.193	−0.011	0.141	−0.207
Cognitive function	0.011	−0.013	−0.031	−0.123
Work status	0.072	0.230	0.467***	−0.089
Burden of kidney disease	0.025	0.059	0.116	0.099
Effects of kidney disease	0.016	0.166	−0.090	−0.072
List of symptoms/problems	−0.233	0.036	−0.136	0.029
KDQOL-SF 36				
Mental health	0.161	0.269	0.149	0.121
Limitations due to emotional health problems	−0.231	−0.146	−0.019	−0.018
Social functioning	0.096	0.051	0.165	0.266
Energy/Fatigue	0.229	0.354	0.282	0.044
Pain	0.075	0.011	−0.493**	0.335
Perception of general health	0.103	0.022	0.044	0.233
Physical Functioning	−0.158	−0.254	0.084	−0.370*
Limitations due to physical health problems	−0.120	−0.006	0.197	−0.047

Data are reported as Spearman’s correlation coefficient (rho). BMI: body mass index; KDQOL-SF 36: kidney disease quality of life short form 36 questionnaire. *P<0.05; **P<0.01; ***P<0.001.

The association between inflammatory markers and dialysis adequacy parameters is described in Table 5. Hcy was correlated with serum creatinine (P=0.002), GFR (P=0.003), and dialysis time (P=0.02).

**Table 5.** Spearman’s correlation coefficients between inflammatory markers and dialysis adequacy parameters.

Inflammatory markers	Dialysis adequacy parameters						
	Kt/v	Urea	Creatinine	GFR	Number of fistulas	Number of catheters	Dialysis time
TNF-α	0.203	−0.155	−0.155	0.113	0.031	0.136	−0.079
IL-6	−0.132	−0.067	−0.180	0.123	−0.295	−0.024	−0.118
CRP	−0.163	0.015	0.150	−0.176	−0.199	0.09	−0.401*
Hcy	−0.175	0.257	0.538**	−0.518**	0.173	0.149	0.411*
Ferritin	−0.115	0.047	0.126	−0.170	−0.051	0.217	0.145

Data are reported as Spearman’s correlation coefficient (rho). GFR: glomerular filtration rate; TNF-α: tumor necrosis factor alpha; IL-6: interleukin 6; CRP: ultra-sensitive C-reactive protein; Hcy: homocysteine. *P<0.05; **P<0.01.

Among the inflammatory markers and nutritional parameters, there was a correlation between TNF-α and albumin (P=0.008), nutritional status (P=0.04), and arm circumference adequacy (P=0.01). TNF-α was the inflammatory marker most commonly associated with nutritional parameters. There was an association between ferritin and nutritional status (P=0.03), and between IL-6 and arm circumference adequacy (P=0.02; Table 6).

**Table 6.** Spearman’s correlation coefficients between inflammatory markers and nutritional parameters.

Inflammatory markers	Nutritional parameters			
	Albumin	Nutritional status	Triceps skinfold adequacy	Arm circumference adequacy
TNF-α	−0.474*	0.361*	0.371*	−0.128
IL-6	−0.424*	0.224	0.353	−0.002
CRP	−0.375*	0.419*	0.490**	0.308
Hcy	0.079	0.178	−0.194	0.200
Ferritin	−0.162	0.388*	−0.131	0.153

Data are reported as Spearman’s correlation coefficient (rho). TNF-α: tumor necrosis factor alpha; IL-6: interleukin 6; CRP: ultra-sensitive C-reactive protein; Hcy: homocysteine. *P<0.05; **P<0.01.

There was no association between nutritional adequacy parameters and albumin with triceps skinfold thickness adequacy (P=0.66), with arm circumference adequacy (P=0.92), and with BMI (P=0.37).

## Discussion

CKD is commonly accompanied by a negative impact on patient perception of health. These factors may also reflect in other aspects such as nutrition, cognition, and psychosocial factors (11). The KDQOL-SF36 is an instrument for evaluating the perception of quality of life in patients with end-stage renal disease and has a score ranging from 0 to 100, allowing subjective measurement of important variables for proper treatment follow-up (12).

Patients on hemodialysis commonly present weight gain during the interdialytic period (13). Our data demonstrated that 53.4% of the patients studied had an interdialytic weight gain of 3 kg; 36.6% gained from 3 to 5 kg and 10% had a weight gain of over 5 kg. In our evaluation, we found no correlation between weight gain and the other quality of life parameters as well as with inflammatory markers.

When evaluating the nutritional profile and weight gain of the individuals studied, we found that 3.4% of the patients were malnourished and 46.6% were classified as eutrophic. On the other hand, 40% were overweight and 10% were obese. Malnutrition apparently was not a predictive factor for worse quality of life in this population (14,15).

Ferraz et al. (16), studying the nutritional status in patients submitted to hemodialysis, found a correlation between malnutrition and interdialytic weight gain. However, they emphasized that findings should not be generalized due to interferences of individual factors that may reflect on nutrition and weight.

Tannor et al. (17), in an evaluation of the quality of life in dialysis patients in South Africa, found that patients on dialysis have a poor quality of life regardless of the treatment being peritoneal dialysis or hemodialysis. The parameters that differentiated the analysis were scores of specific domains for each type of treatment, but in general, both therapies resulted in reduction of quality of life.

Almutary et al. (18) demonstrated that sexual dysfunction affects approximately one third of dialytic renal patients and is recurrent mainly in older individuals. On the other hand, it has been reported that dialysis patients have difficulty in feeling comfortable and having a satisfactory sex life due to the use of catheters and the discomfort caused by generalized pain (17). Thus, we believe that the pain variable is one of the preponderant factors that interferes in the other variables of quality of life perception.

Raghavan et al. (19) state that pain is a symptom that increases in parallel with the progression of kidney disease and grows exponentially as the patient ages. The authors suggest that these patients initiate pain-supportive care that does not affect renal disease progression. Moreover, pain may also reflect a limitation to perform essential activities, as verified in the parameter “limitations due to physical health problems” (6–11).

The parameters with the best indices were: the “effects of kidney disease”, “list of symptoms/problems”, “sleep”, and the “perception of the burden of kidney disease”. The variables “cognitive function”, “limitations due to emotional health problems”, and “social functioning” obtained the lowest scores. Studies have suggested that one of the possible determinants of cognition loss in these patients is the decline in GFR and increased synthesis of proinflammatory mediators (1,20–22).

A study in a rat model of chronic renal failure by 5/6 nephrectomy found an elevation of proinflammatory cytokines. The authors suggest that this increase is related to the decrease in cognition in CKD due to the detection of proinflammatory cytokines in specific nuclei of the central nervous system (23).

Our study found that Hcy was correlated with the “work status” variable and IL-6 levels with the variable “limitations due to health problems”. Hcy participates in the physiopathology of cardiovascular diseases (23), Alzheimer's (24), depression (25), and in kidney disease (26). All of these pathologies are directly involved in loss of quality of life and limitations due to these health problems, which may directly reflect on performance at work and other social activities.

When analyzing the relationship between anthropometric data and the same inflammatory mediators, we found that there was a significant correlation between abdominal circumference and ferritin, and between BMI and both CRP and ferritin values. “Triceps skinfold thickness” was correlated with values of TNF-α. There is no data in the literature on the relationship between measurements of abdominal circumference or BMI and ferritin levels in end-stage renal patients. It is possible that the nutritional status of the studied population directly influenced this interaction.

There is evidence indicating the participation of hyperhomocysteinemia in the pathophysiology of kidney disease (26). Foucher et al. (27), reporting the relationship between Hcy and serum creatinine levels, showed that the increase in Hcy is much greater than the elevation of creatinine, making it clear that Hcy alterations progress more rapidly than alterations in serum creatinine levels in kidney patients. The authors also report that these factors may contribute significantly to an increased mortality rate and they attribute this to the increased release of inflammatory mediators.

Although the observed results contribute to future assessments of quality of life in patients during hemodialysis treatment, it should be noted that the number of patients studied may not allow generalization of the findings. In addition, a one-time blood sample from patients treated in a single center may limit the generalizability and reliability of the results. Multiple hypotheses were tested, and the small sample size did not allow correcting P-values. Therefore, our study may have a high probability for global type I error.

In conclusion, Hcy was directly related to nutritional status, creatinine, and inflammatory markers. In the studied population, most sections of the KDQOL-SF36 were not correlated with inflammatory cytokine levels.
